# The effect of ALDH2 *rs671* gene mutation on clustering of cardiovascular risk factors in a big data study of Chinese population: associations differ between the sexes

**DOI:** 10.1186/s12872-020-01787-5

**Published:** 2020-12-04

**Authors:** Danchen Wang, Yutong Zou, Songlin Yu, Songbai Lin, Honglei Li, Yicong Yin, Ling Qiu, Tengda Xu, Jie Wu

**Affiliations:** 1 Department of Laboratory Medicine, Peking Union Medical College Hospital, Chinese Academy of Medical Sciences, No. 1 Shuaifu Yuan, Dongcheng District, Beijing, 100730 China; 2 Department of Health Care, Peking Union Medical College Hospital, Chinese Academy of Medical Sciences, No. 1 Shuaifu Yuan, Dongcheng District, Beijing, 100730 China

**Keywords:** *ALDH2*, rs671, Cardiovascular disease, Cardiovascular risk factor

## Abstract

**Background:**

The *ALDH2* rs671 genetic polymorphism has been linked with cardiovascular diseases (CVDs), but comprehensive epidemiological studies are lacking. An observational, retrospective big data study was carried out to evaluate the associations between this polymorphism and clustering cardiovascular risk factors (CRFs) in a Chinese population.

**Methods:**

A total of 13,101 individuals (8431 males and 4670 females) were enrolled. Genetic polymorphism was assessed using gene mutation detection kits, coupled with an automatic fluorescent analyzer. Other data were obtained from the records of the Department of Health Care at Peking Union Medical College Hospital.

**Results:**

Comparing the concentrations of common biochemical analytes, including BMI, SBP, DBP, ALT, AST, γ-GT, TBil, Cr, Glu, TC, TG, and HDL-C among individuals with the GG, GA, and AA genotypes of *ALDH2* rs671, we found significant differences in males (all *p* < 0.001), but not in females. For males, the frequencies of hypertension, diabetes, and obesity were significantly higher for GG than for GA or AA (all *p* < 0.05). However, there was no significant difference for dyslipidemia, and no significant associations were observed for all frequencies in females. The prevalence of individuals with 1–4 CRFs was significantly higher among GG males than those carrying GA or AA, and fewer GG males had non-CRFs (all *p* < 0.05).

**Conclusion:**

Polymorphisms of *ALDH2* rs671 are associated with clustering CRFs, especially hypertension and diabetes in males, but not in females. These associations are likely mediated by alcohol intake, which is also associated with this gene.

## Background

Cardiovascular diseases (CVDs) is one of the leading causes of mortality worldwide [1, 2], with more than 55 million deaths caused by CVDs in 2017 [3]. Hypertension, diabetes, obesity, and dyslipidemia are well known as cardiovascular disease risk factors (CRFs) [4–7]. CRFs are common, carry an increased risk of CVDs, and their prevalence increases with age [1]. Moreover, the effect of clustering CRFs is greater than the effect of single CRFs on the same individual [2].

Alcohol is one of the most widely used recreational substances worldwide, and its intake is a leading risk factor for global disease burden, including CVDs [4–7]. Despite general recognition that alcohol intake has a negative effect on health, it has been estimated that the average ethanol consumption of a person aged more than 15 years is approximately 19.7 mL per day [8]. Other data suggest that global adult per-capita consumption is estimated to increase from 6.5 L (95% CI: 6.0 l–6.9 L) in 2017 to 7.6 L (95% CI: 6.5–10.2 L) by 2030 [9].

As an essential bioactivating enzyme, ALDH2 can degrade acetaldehyde to nontoxic acetic acid. It is encoded by the *ALDH2* gene, which is commonly polymorphic in East Asian populations [5]. It has been reported that as many as 30–50% of East Asians carry an inactive form of ALDH2-rs671 resulting from a single G-to-A transition causing replacement of glutamate to lysine at position 504, and drastically reducing the carrier’s capacity to metabolize alcohol [10–12]. The frequency of the A allele was reported to be 0.21 in China [13].

ALDH2 activation has also been found to be associated with improved mitochondrial function and the remodeling of ventricular function [14, 15], and many studies have reported an association between ALDH2 and CVDs [1, 2, 5, 13, 15, 16]. The most important known feature of the myocardial cardio-protective role of ALDH2 is the clearance of toxic aldehydes such as 4-hydroxynonenal and its adducts, which can be induced by acute oxidative stress upon cardiac ischemia or reperfusion [17–19]. Activation of ALDH2 may slow down the progression of atherosclerosis via attenuation of endoplasmic reticulum stress and apoptosis in smooth muscle cells [16].

Genetic association studies have recently shown that the *ALDH2* rs671 polymorphism is a significant risk factor for hypertension, diabetes, and coronary heart diseases in Asian people [20, 21]. Although a number of studies have focused on the association between *ALDH2* and single CRFs such as hypertension, diabetes, obesity, and dyslipidemia, and analyses [20, 21], the association has not been clearly defined. Thus, detailed studies focused on the association between *ALDH2* and clustering CRFs are needed. Interestingly, there is an increasing interest in obtaining annual routine physical examination in China, which has resulted in more data on the health status of the population. Using data from hospital and laboratory information systems is not only cost-effective but also efficient.

Therefore, this retrospective study, which is based on clinical big data, aimed to (1) evaluate the distribution of *ALDH2* rs671 genotypes, (2) evaluate the prevalence of single and clustering CRFs in China, and (3) explore the association between *ALDH2* rs671 genotypes and CRFs.

## Methods

### Data collection

The study included 13,101 patients aged ≥19 years old. Data including demographic information, common biochemical analytes, and medical history from November, 2013 to October, 2018, were obtained from the hospital information system (HIS) and laboratory information system (LIS) of the Department of Health Care at Peking Union Medical College Hospital (PUMCH). With a unique identification code identifying duplicated measurements, only the first record of each person was saved.

### Laboratory measurement

Genomic DNA was extracted from whole peripheral blood via DNA extraction kits (Tianlong Technology Co. LTD, Xi’an, China) and rs671 polymorphism status was determined by an *ALDH2* gene mutation detection kit, coupled with an automatic fluorescent analyzer (Beijing market gene technology Co. LTD, Beijing, China). Height, weight, and blood pressure were measured by well-trained nurses and doctors, and body mass index (BMI) was calculated as weight divided by height squared. Common biochemical analytes including Albumin (Alb), alanine aminotransferase (ALT), Aspartate aminotransferase (AST), glutamyl transpeptidase (γ-GT), total bilirubin (TBil), creatinine (Cr), glucose (Glu), total cholesterol (TC), triglyceride (TG), high density lipoprotein cholesterol (HDL-C), and low density lipoprotein cholesterol (LDL-C) were measured by a Roche C8000 automatic analyzer (Roche C8000, Basel, Switzerland) with corresponding reagents, calibrators, and quality control materials. All records including quality control and external quality assessment during this period were reviewed and deemed sound.

### Definition of cardiovascular disease risk factors (CRFs)

In this study, we evaluate the association between the *ALDH2* rs671 polymorphism and major CRFs including hypertension, diabetes, obesity, and dyslipidemia. We used the following specific definitions, as previously described [22]:

1. Hypertension: systolic blood pressure (SBP) ≥140 mmHg and/or diastolic blood pressure (DBP) ≥90 mmHg [23].
2. Diabetes: fasting blood Glu ≥7 mmol/L or HbA1C ≥6.5%.
3. Obesity: BMI ≥28 kg/m^2^.
4. Dyslipidemia: at least one of the following: TC ≥5.2 mmol/L, TG ≥1.7 mmol/L, HDL-C < 1.0 mmol/L, and/or LDL-C ≥ 3. 4 mmol/L.

### Statistical analysis

Excel 2010 (Microsoft Inc., USA), SPSS 20.0 software (SPSS Inc., Chicago, IL, USA), and Graphpad prism for Windows (GraphPad Software, San Diego, CA), were used for our statistical analyses. The Mann-Whitney U or Kruskal-Wallis tests were used to compare measurements among groups, and the comparisons of prevalence were conducted by Chi-square test. Multivariate logistic regression analysis was used to correct for covariates and calculate the odds ratios (ORs), with 95% confidence intervals (CIs), of genotype associations with CRFs. The results were considered statistically significant when the two-sided *p*-value was < 0.05.

## Results

### Basic characteristics of the studied population

The baseline demographic and clinical characteristics of studied individuals divided by *ALDH2* polymorphism and sex are shown in Table 1. In total, 13,101 individuals including 8431 males and 4670 females were eventually included. The distribution of age was (49 ± 9) years old, and BMI was (24.8 ± 3.8) kg/m^2^. There was no difference in age by *ALDH2* polymorphism in either males or females. However, common clinical measurements including BMI, SBP, DBP, ALT, AST, γ-GT, TBil, Cr, Glu, TC, TG, and HDL-C were significantly different in males (all *p* < 0.001), though not in females.

**Table 1:** General characteristics of the enrolled population

	GG		GA		AA		*P* in male	*P* in female
	Male (n=5636)	Female (n=3255)	Male (n=2571)	Female(n=1286)	Male (n=224)	Female (n=129)		
Age (years)	48 ± 9	49 ± 9	49 ± 9	48 ± 9	48 ± 8	48 ± 9	0.795	0.509
BMI (kg/m2)	25.8 ± 3.6	23.4 ± 3.3	25.4 ± 3.7	23.1 ± 3.7	25.2 ± 4.1	23.3 ± 3.7	< 0.001	0.075
SBP (mmHg)	125.2 ± 15.1	116.3 ± 16.5	122.5 ± 14.7	116.1 ± 18.1	119.5 ± 13.8	117.2 ± 20.2	< 0.001	0.395
DBP (mmHg)	79.2 ± 10.5	69.2 ± 10.1	76.8 ± 9.9	68.5 ± 10.5	74.7 ± 10.0	70.4 ± 11.8	< 0.001	0.060
Alb (g/L)	62.8 ± 2.9	60.6 ± 2.9	62.9 ± 2.9	60.7 ± 2.9	62.6 ± 3.3	61.1 ± 2.9	0.460	0.182
ALT (U/L)	25 (18, 34)	16 (12, 21)	21 (16, 31)	15 (12, 21)	23 (18, 31)	16 (12, 22)	< 0.001	0.916
AST (U/L)	20 (17, 25)	18 (15,21)	19 (16, 23)	18 (15,21)	19 (17, 23)	17 (15, 21)	< 0.001	0.273
γ-GT (U/L)	36 (24, 60)	16 (12, 23)	28 (20, 43)	16 (12, 23)	24 (18, 35)	15 (12, 25)	< 0.001	0.849
Tbil (μmol/L)	11.9 (9.2, 15.3)	9.0 (6.9, 11.9)	11.2 (8.7, 14.5)	8.9 (7.0, 11.5)	10.3 (8.0, 13.9)	9.3 (6.9, 11.7)	< 0.001	0.743
Cr (μmol/L)	79.8 ± 13.4	60.4 ± 10.0	81.6 ± 12.4	61.3 ± 22.8	83.0 ± 11.3	59.8 ± 8.7	< 0.001	0.546
Glu (mmol/L)	5.7 ± 1.6	5.2 ± 1.1	5.5 ± 1.4	5.2 ± 1.0	5.4 ± 1.2	5.2 ± 1.3	< 0.001	0.904
TC (mmol/L)	4.82 ± 0.98	4.89 ± 0.92	4.69 ± 0.88	4.88 ± 0.92	4.67 ± 0.85	4.88 ± 1.00	< 0.001	0.890
TG (mmol/L)	1.6 (1.2, 2.4)	1.1 (0.8, 1.6)	1.5 (1.1, 2.2)	1.1 (0.8, 1.6)	1.4 (1.0, 2.1)	1.2 (0.8, 1.6)	< 0.001	0.644
HDL-C (mmol/L)	1.11 ± 0.27	1.39 ± 0.33	1.08 ± 0.25	1.38 ± 0.34	1.05 ± 0.23	1.35 ± 0.34	< 0.001	0.398
LDL-C (mmol/L)	3.03 ± 0.81	3.05 ± 0.79	3.00 ± 0.76	3.03 ± 0.79	3.03 ± 0.74	2.96 ± 0.90	0.088	0.243

### *ALDH2* rs671 genotype frequency by sex and age

**Fig. 1:**
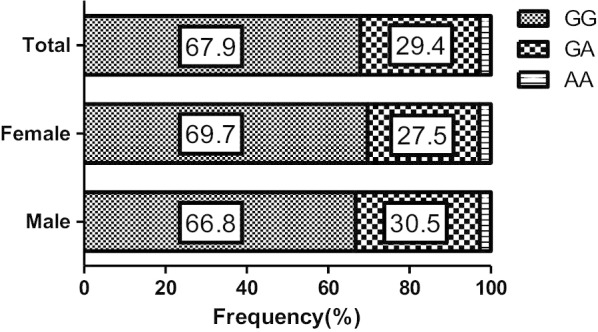
The frequency of ALDH2 rs671 genotype by sex

The distribution of *ALDH2* rs671 gene polymorphism among different years (from 2013 to 2018) did not show significant differences (*p* = 0.946). As Fig. 1 and Supplemental Table 1 show, the frequencies of the *ALDH2* rs671 genotypes GG, GA, and AA in the total population were 67.9, 29.4, and 2.7%, respectively. These frequencies did not differ significantly by sex. Although there was no significant difference of the overall age distribution of the different genotypes in either males or females, the frequency of AA in individuals aged ≥65 years old was lower than other age groups in both males and females, with the opposite distribution in evidence for GG. Also, the frequency of GA in those aged between 19 and 29 years was higher than in other age groups, and the frequency of GG was significantly lower (Supplemental Table 1).

### Prevalence of CRFs by rs671 genotype

The frequencies of CRFs associated with different rs671 genotypes by sex are shown in Table 2. For males, the frequencies of hypertension, diabetes, and obesity were significantly higher for GG than for GA or AA. However, there was no significant difference in the prevalence of dyslipidemia among the three rs671 genotypes. For females, there was no statistically significant difference in the prevalence of hypertension, diabetes, obesity, or dyslipidemia among the rs671 genotypes (all *p* > 0.05).

**Table 2:** Prevalence of CRFs by rs671 genotype and sex

CRFs	GG		GA		AA		*P* in male	*P* in female
	Male	Female	Male	Female	Male	Female		
Hypertension	2662 (47.2%)	621 (19.1%)	992 (38.6%)	245 (19.1%)	66 (29.5%)	34 (26.4%)	< 0.001	0.118
Obesity	1320 (23.4%)	276 (8.5%)	501 (19.5%)	113 (8.8%)	42 (18.8%)	15 (11.6%)	< 0.001	0.450
Diabetes	791 (14.0%)	170 (5.2%)	279 (10.9%)	78 (6.1%)	26 (11.6%)	10 (7.8%)	< 0.001	0.284
Dyslipidemia	4030 (71.5%)	1614 (49.6%)	1807 (70.3%)	655 (50.9%)	152 (67.9%)	63 (48.8%)	0.300	0.693

### Prevalence of clustering CRFs by rs671 polymorphism

The non-CRFs were defined as individuals who did not have hypertension, obesity, diabetes, or dyslipidemia. The frequencies of non-CRFs were 15.3, 40.9, and 24.4% in males, females, and the total population. The respective frequencies of individuals with one, two, three, and four CRFs were 36.9, 32.4, 12.8, and 2.5% in males, 40.0, 14.7, 3.9, and 0.6% in females. The major cluster of CRFs comprised hypertension, diabetes, obesity, and dyslipidemia. The frequencies of clustering CRFs by rs671 genotype and sex are shown in Table 3. The sex-stratified frequencies of clustered CRFs among the rs671 genotypes were significantly different in males, but not in females. The frequencies of individuals with two, three, and four CRFs were significantly higher in the population with GG than in those with GA or AA in males, while among males with no CRFs, the frequency of GG was statistically lower than GA or AA. However, there was no significant difference between the frequencies of clustering CRFs and *ALDH2* genotype in females.

**Table 3:** Prevalence of clustering CRFs by rs671 genotype and sex

CRFs	GG		GA		AA		*P* in male	*P* in female
	Male	Female	Male	Female	Male	Female		
CRF = 0	797 (14.1%)	1335 (41.0%)	449 (17.5%)	521 (40.5%)	42 (18.8%)	52 (40.3%)	< 0.001	0.946
CRF = 1	1993 (35.4%)	1311 (40.3%)	1019 (39.6%)	513 (39.9%)	101 (45.1%)	42 (32.6%)	< 0.001	0.214
CRF = 2	1895 (33.6%)	478 (14.7%)	782 (30.4%)	185 (14.4%)	58 (25.9%)	25 (19.4%)	0.002	0.309
CRF = 3	782 (13.9%)	110 (3.4%)	276 (10.7%)	60 (4.7%)	19 (8.5%)	10 (7.8%)	< 0.001	0.008
CRF = 4	168 (3.0%)	21 (0.6%)	41 (1.6%)	7 (0.5%)	3 (1.3%)	0 (0%)	0.001	0.622

### Multivariate logistic regression analysis

Multivariate logistic regression analysis results are shown in Table 4. This analysis estimated OR with 95% CI for each variable, while adjusting for age and other risk factors. Compared with GG, males with GA and AA were less likely to have hypertension (GA: OR = 0.77, 95% CI: 0.69–0.85; AA: OR = 0.56, 95% CI: 0.41–0.75). Also, males with GG were more likely to have diabetes than those with GA (OR = 0.73, 95% CI: 0.62–0.87). There were no differences in overweight or dyslipidemia among male populations with GG, GA, and AA. For females, there was no significant difference among genotypes in hypertension, diabetes, obesity, or dyslipidemia. In males, though not in females, the proportions of GA and AA decreased with increasing numbers of CRFs.

**Table 4:** Multivariate logistic regression analysis

CRFs	GG	GA			AA		
		OR	LL	UL	OR	LL	UL
Male							
Hypertension	1(ref)	**0.77**	0.69	0.85	**0.56**	0.41	0.75
Diabetes	1(ref)	**0.73**	0.62	0.87	**0.79**	0.47	1.32
Overweight	1(ref)	**0.88**	0.74	1.05	**0.95**	0.56	1.59
Dyslipidemia	1(ref)	**1.05**	0.94	1.16	**1.00**	0.75	1.34
CRFs = 1	1(ref)	**0.95**	0.83	1.08	**0.93**	0.66	1.31
CRFs = 2	1(ref)	**0.72**	0.63	0.83	**0.46**	0.31	0.69
CRFs = 3	1(ref)	**0.59**	0.48	0.73	**0.40**	0.21	0.77
CRFs = 4	1(ref)	**0.40**	0.22	0.71	**0.57**	0.13	2.38
Female							
Hypertension	1(ref)	**1.04**	0.87	1.24	**1.55**	1.01	2.40
Diabetes	1(ref)	**1.17**	0.83	1.66	**1.41**	0.61	3.25
Overweight	1(ref)	**1.06**	0.75	1.49	**1.57**	0.72	3.41
Dyslipidemia	1(ref)	**1.12**	0.97	1.28	**0.97**	0.67	1.41
CRFs = 1	1(ref)	**1.06**	0.92	1.23	**0.95**	0.62	1.45
CRFs = 2	1(ref)	**1.02**	0.82	1.27	**1.88**	1.12	3.16
CRFs = 3	1(ref)	**1.71**	1.13	2.59	**1.54**	0.52	4.60
CRFs = 4	1(ref)	**1.04**	0.27	3.97	**Non**	Non	Non

## Discussion

Based on the distribution of age, the enrolled individuals fairly reflected the distribution of Chinese adults, the frequency of GA, AA and AA during the whole 5 years was 29.4, 2.7 and 17.4%, similar to those of previous studies [13, 24]. The distribution of *ALDH2* rs671 gene polymorphism among different years (from 2013 to 2018) did not show significant differences (*p* = 0.946), which implied the reliability of the measurements without obvious carry-over.

ALDH2 activation, which plays key roles in clearing toxic aldehydes, improving mitochondrial function, and remodeling ventricular function, has been shown to be protective against the development of CVDs [14–19, 25], suggesting that *ALDH2* gene mutation should be harmful for human health. However, the results of clinical trials have been inconsistent, with many of them indicating a protective effect of the A allele against hypertension, dyslipidemia, and diabetes [3, 4, 21, 26]. In this study, we found that the A allele may be more likely to be protective against clustering CRFs, especially hypertension and diabetes in males, though not in females. The contradictory results between basic research and clinical studies, and between males and females, could be explained by the influence of lifestyles, especially the amount and pattern of alcohol consumption. A study based on the China Kadoorie Biobank reported that 33% of males drank alcohol in most weeks, mainly as spirits, while only 2% of females did so [13]. Because of issues with alcohol tolerance, including uncomfortable feelings such as flush, dizziness, vomiting, and even exhaustion, individuals carrying the A allele, especially those with the AA genotype, usually drink less (GG: 157 g/week; AG: 37 g/week; AA: 3 g/week) [13]. Furthermore, alcohol intake has been found to be closely associated with an increased risk of CVDs [4–7], and reducing alcohol intake can lower blood pressure in a dose-dependent manner [25]. Therefore, it is very likely that the influence of the different *ALDH2* rs671 gene polymorphisms on the prevalence of CRFs is substantially mediated by the amount and pattern of alcohol consumption. Interestingly, we also found that the frequency of AA in individuals ≥65 years old was lower than in other age groups, especially 18–29, with *p* = 0.01, which may imply that the *ALDH2* rs671 mutation can induce other mortal diseases and aging independently of CVDs [26, 27].

In this study, we found that, compared with GG carriers, males with GA and AA were less likely to have hypertension. Our results are consistent with a case control study which found that those carrying the A allele were at a lower risk of essential hypertension in males [AA/AG vs. GG: OR (95% CI) = 0.76 (0.58–0.98)], but not in females [21]. However, our results are contrary to a cross-sectional study, which found that the individuals with the rs671 A allele were at higher risk for the development of essential hypertension [28]. In that study, the association was not evaluated separately for males and females, and based on our data, that could have substantially influenced the results. Moreover, our data on the relationship between *ALDH2* rs671 genotype and the distributions of TC, TG, and HDL-C, are consistent with previous studies [4, 26, 29]. However, the relationship between rs671 and the prevalence of dyslipidemia as such was not recognized in those studies [4, 26, 29]. Also, we found that the individuals with the rs671 A allele had lower Glu levels and lower prevalence of diabetes, though multivariate logistic regression analysis results didn’t show that the A allele was significantly protective for diabetes in either males or females. This is similar to a previous Mendelian randomization analysis, which showed that the A allele in males was significantly associated with decreased diabetes risk for both the overall population (OR = 0.716, 95% CI: 0.567–0.904, *p* = 0.005) and moderate drinkers (OR = 0.564, 95% CI: 0.355–0.894, *p* = 0.015) [30]. Interestingly, another study found that the individuals with the A allele had a lower incidence of microvascular complications associated with alcohol consumption, but a higher incidence of macrovascular complications irrespective of alcohol consumption [31]. This also implied that the incidence of CRFs could be mediated by both genetics and lifestyle factors such as alcohol assumption.

Although there have been many other studies exploring and evaluating the association between *ALDH2* genotype and many diseases including CVDs and their risk factors, most of them were animal experiments. Epidemiological studies did not emerge until recently, and most have focused on the association between *ALDH2* and single CRFs, rather than clustering CRFs. In this study, we derived clinical big data from the HIS and LIS of PUMCH, which was simple, cost-efficient and a good reflection of the general population. With all individuals represented in PUMCH being analyzed over a five-year period by the same analytical systems, variation due different methods or facilitates was avoided, and the demographic information and clinical laboratory measurements were thorough. Furthermore, we were able to analyze hypertension, diabetes, obesity, and dyslipidemia simultaneously, while correcting for covariations via multivariate logistic regression analysis.

However, some limitations of this study are notable. Alcohol intake was not considered in the evaluation, and other important factors such as smoking and socioeconomic situation were also lacking. Also, in this cross-sectional study, the major CRFs, including hypertension, diabetes, obesity, and dyslipidemia, were assessed based only on single test of the corresponding clinical measurements. Casual inferences from this study should therefore be avoided. In the future, long term follow-up cohort studies considering more details, especially the pattern of alcohol consumption, are needed to further explore the causal relationships suggested by our data.

## Conclusion

Our study indicates that the *ALDH2* gene polymorphism is associated with clustering CRFs, and that the rs671 A allele may be protective against clustering CRFs in males. This is likely mediated by alcohol intake or related lifestyle factors associated with this genetic variant.

## Supplementary information

**Additional file 1: Supplemental Table 1.** The frequency of ALDH2 rs671 genotype by age and sex.

## Data Availability

The datasets generated and analysed during the current study are available from the corresponding author on reasonable request. The datasets generated and/or analysed during the current study are available in the National Center for Biotechnology Information repository, https://www.ncbi.nlm.nih.gov/gene/217.

## Abbreviations

CVDs: Cardiovascular diseases
CRFs: Cardiovascular disease risk factors
ALDH2: Aldehyde dehydrogenase 2
HIS: Hospital Information System
LIS: Laboratory Information System
PUMCH: Peking Union Medical College Hospital
BMI: Body mass index
Alb: Albumin
ALT: Alanine aminotransferase
AST: Aspartate aminotransferase
TBil: Total bilirubin
Cr: Creatinine
Glu: Glucose
TC: Total cholesterol
TG: Triglyceride
HDL-C: High density lipoprotein cholesterol
LDL-C: Low density lipoprotein cholesterol
SBP: Systolic blood pressure
DBP: Diastolic blood pressure
OR: Odds ratio
CI: Confidence interval
