# Supplementary material for: The effect of ALDH2 rs671 gene mutation on clustering of cardiovascular risk factors in a big data study of Chinese population: associations differ between the sexes
Source: BMC Cardiovasc Disord. 2020 Dec 4;20:509. doi: 10.1186/s12872-020-01787-5 (PMC7716427; doi:10.1186/s12872-020-01787-5)
Supplement: Supplementary file 1 — Additional file 1: Supplemental Table 1. The frequency of ALDH2 rs671 genotype by age and sex. [file 12872_2020_1787_MOESM1_ESM.docx]

**Supplemental Table 1**

| Age (years) | GG | | | GA | | | AA | | | *P*  in male | *P*  in female |
| --- | --- | --- | --- | --- | --- | --- | --- | --- | --- | --- | --- |
|  | Male | Female | Total | Male | Female | Total | Male | Female | Total |  |  |
| 19-29 | 96  (64.4%) | 43  (51.8%) | 139  (59.9%) | 49  (32.9%) | 37  (44.6%) | 86  (37.1%) | 4  (2.7%) | 3  (3.6%) | 7  (3.0%) | 0.01 | 0.262 |
| 30-39 | 701  (67.5%) | 383  (70.7%) | 1084  (68.6%) | 315  (30.3%) | 145  (26.8%) | 460  (29.1%) | 23  (2.2%) | 14  (2.6%) | 37  (2.3%) | 0.84 | 0.336 |
| 40-49 | 2331  (67.6%) | 1346  (69.5%) | 3677  (68.3%) | 1025  (29.7%) | 531  (27.4%) | 1556  (28.9%) | 93  (2.7%) | 60  (3.1%) | 153  (2.8%) | 0.57 | 0.16 |
| 50-64 | 2287  (65.8%) | 1330  (70.2%) | 3617  (67.4%) | 1086  (31.3%) | 516  (27.2%) | 1602  (29.8%) | 101  (2.9%) | 48  (2.5%) | 149  (2.8%) | 0.81 | 0.093 |
| ≥65 | 221  (69.1%) | 153  (71.5%) | 374  (70.0%) | 96  (30.0%) | 57  (26.6%) | 153  (28.7%) | 3  (0.9%) | 4  (1.9%) | 7  (1.3%) | Ref | Ref |
| Total | 5636  (66.8%) | 4670  (69.7%) | 8891  (67.9%) | 2571  (30.5%) | 1286  (27.5%) | 3857  (29.4%) | 224  (2.7%) | 129  (2.8%) | 353  (2.7%) | NA | NA |
